# Incidence of childhood cancers in the North East geopolitical zone of Nigeria

**DOI:** 10.3389/fonc.2024.1379968

**Published:** 2024-08-30

**Authors:** Dauda Eneyamire Suleiman, Adamu Saidu Adamu, Uchenna Simon Ezenkwa, Maimuna Orahachi Yusuf, Aliyu Ibrahim Lawan, Rufai Abdu Dachi, Kefas John Bwala, Haruna Usman Liman, Abba Kabir, Adamu Isa Adamu, Modu Abubakar Kolomi, Abdulrazaq Ajanaku Jimoh, Ismaila Isa Garba, Yusuf Mohammed Abdullahi, Bala Mohammed Audu

**Affiliations:** ^1^ Department of Histopathology, College of Medical Sciences, Abubakar Tafawa Balewa University, Bauchi, Bauchi State, Nigeria; ^2^ Department of Pediatrics, College of Medical Sciences, Abubakar Tafawa Balewa University, Bauchi, Bauchi State, Nigeria; ^3^ Department of Histopathology, Federal University of Health Sciences, Azare, Bauchi State, Nigeria; ^4^ Department of Pediatrics, Federal University of Health Sciences/Federal Medical Centre, Azare, Nigeria; ^5^ Department of Histopathology, College of Medical Sciences, Gombe State University/Federal Teaching Hospital, Gombe, Gombe State, Nigeria; ^6^ Department of Hematology and Blood Transfusion, College of Medical Sciences, Abubakar Tafawa Balewa University, Bauchi, Nigeria; ^7^ Department of Surgery, College of Medical Sciences, Abubakar Tafawa Balewa University, Bauchi, Nigeria; ^8^ Department of Histopathology, College of Medical Sciences, University of Maiduguri/University of Maiduguri Teaching Hospital, Maiduguri, Borno State, Nigeria; ^9^ Department of Histopathology, Yobe State University/Yobe State University Teaching Hospital, Damaturu, Yobe State, Nigeria; ^10^ Department of Pathology, Federal Medical Centre, Nguru, Yobe State, Nigeria; ^11^ Department of Surgery, Federal Medical Centre, Azare, Nigeria; ^12^ Department of Obstetrics and Gynecology, Federal University of Health Sciences, Azare, Nigeria

**Keywords:** childhood cancer, pediatrics, oncology, Northeast Nigeria, sub-Saharan Africa

## Abstract

**Introduction:**

Cancers are a major cause of childhood mortality worldwide especially in LMICs where underdiagnoses and lack of quality cancer data hampers effective cancer control efforts. This study aimed to document and describe the patterns and characteristics of childhood cancers in the North East geopolitical zone of Nigeria.

**Methods:**

This was a retrospective cross-sectional study that collected cancer data from 4 out of the 6 states in the North East of Nigeria. The data included all malignancies diagnosed in children aged 0-19 years between 2019 and 2022. The age-specific incidence rates were also calculated for the individual 5-year age groups (0–4 years, 5–9 years, 10–14 years, and 15–19 years). The crude incidence rates (CIR) were calculated as the weighted averages of the respective ASRs in each age range within 0-14 years and 0-19 years respectively. The cancers were grouped according to the International Incidence of Childhood Cancers, volume 3 (IICC3).

**Results:**

Cancers in people <20 years accounted for 7.3% of all cancers diagnosed over the same period. The crude incidence rates (CIR) for cancers in children and adolescents were 20.9 per million children aged 0-19 years and 18.8 per million children aged 0-14 years respectively, while the age-standardized rates (ASR) were 1.80 and 1.63 per million person-years respectively. There was a variation in the most commonly diagnosed cancers across all age groups. However, lymphomas were the most commonly diagnosed cancers overall, while CNS tumors were overwhelmingly rare.

**Conclusion:**

Despite data limitations, this study provides useful insights into patterns of cancers in the region and will hopefully provide a basis for the strengthening of pediatric oncology care, childhood cancer control programs and population-based cancer registries.

## Introduction

Cancers are a major cause of morbidity and mortality in children and adolescents worldwide with an estimated 400, 000 children and adolescents being afflicted by cancer annually (1–3). The relative rarity of cancers in children compared to adults places a lot of psychosocial concerns on parents and caregivers when these cancers are diagnosed. Childhood cancer figures may be much higher than available data suggests, especially in low- and middle-income countries (LMICs) where a lack of advanced diagnostic facilities and the presence of more prevalent infectious and nutritional diseases may result in missed diagnoses (4). The latter scenario is particularly true for leukemia in childhood, where fever due to superimposed infection is a common mode of presentation, resulting in confusions with more common infectious processes (4, 5). In sub-Saharan Africa, about 90% of children diagnosed with cancer each year are likely to die from the disease, in stark contrast to high-income nations where a roughly 85% overall survival rate is the norm, mainly due to the availability of advanced diagnostic and therapeutic facilities (6).

Comprehensive cancer data are also generally lacking in LMICs and this dearth of data may be worse for the relatively rare childhood cancers (7). Most available data come from the few hospital- and population-based cancer registries. Unfortunately, some of the data available in these registries are incomplete (3). These paucity of quality and reliable data may be one of the contributory factors to the relative neglect of pediatric cancers in cancer control efforts (7). Quality data on pediatric cancers may assist in the prioritization of health resources and the formulation of relevant policies and control programs related to childhood cancer control (7).

Childhood cancers are a heterogenous group of tumors occurring between the age range 0-14 years. However, in the publication of the International Incidence of Childhood Cancer, volume 3 (IICC-3), the target age range was extended to 0-19 years to capture the transition period between childhood and adulthood (2). In general, childhood cancers differ from the adult counterparts in several respects, including biologic behavior, clinical presentation, therapeutic options and treatment outcomes (4). The classification of childhood cancers is mainly based on morphology and topography and on this basis, the 3^rd^ edition of the International Classification of Childhood Cancers (ICCC-3) organizes the various cancer types occurring in childhood into 12 main diagnostic categories (8).

To the best of our knowledge, this study will be the first attempt at a comprehensive documentation of pediatric cancers in the North-East Region of Nigeria and will hopefully provide a basis for awareness creation and policy formulation against these cancers in various states of the region.

## Methodology

### Study area

The North East geopolitical zone of Nigeria consists of six (6) states namely: Adamawa Bauchi, Borno, Gombe, Taraba and Yobe. The population estimates based on the 2006 data indicates that the region accounts for 13.5% of the total population of Nigeria (roughly 19 million people) (9, 10). By 2022, the population of the region is expected to be around 30 million, based on projected annual population growth rate of 2.5% per annum (10).

### Study design

This is a retrospective, descriptive study that collected data on all pathologically confirmed cancers occurring in children and adolescents aged 0-19 years in the tertiary centers of the North East of Nigeria. These centers were selected conveniently as they represent the centers for the diagnosis and management of cancers in the region. The participating centers are six (6) referral tertiary centers located in four out of the six states of the region. These hospitals include the Abubakar Tafawa Balewa University Teaching Hospital (ATBUTH), Bauchi and Federal Medical Centre Azare (FMCA), both in Bauchi State; University of Maiduguri Teaching Hospital (UMTH), Borno State; Federal Teaching Hospital Gombe (FTHG), Gombe State; Federal Medical Centre Nguru (FMCN), and Yobe State University Teaching Hospital (YSUTH), Damaturu, both in Yobe State. Data from two tertiary centers in the Northeast geopolitical zone- Modibbo Adama University Teaching Hospital (MAUTH). Yola and Federal Medical Centre (FMC), Jalingo, was unavailable at the time of this study.

The data sources were from the hospital-based cancer registries and pathology departmental registers for cancers in these hospitals. Data collected include basic biodata, topography/site and pathological diagnosis.

Population distribution data by age and sex for the participating states were extracted from the Nigeria National Population Commission data of 2020 (**Supplementary Table 1**) which contained population projections for the year 2022 (11).

### Data analysis

The various cancers were categorized and sub-categorized according to the ICCC-3 (8). Descriptive statistical analysis was applied on the data obtained using the statistical package for social sciences (SPSS) version 20 (12). The output was categorized into cancer sites by proportions and presented as texts, tables and figures (charts and box and whisker plots). Statistical tests of significance were carried out where necessary and p-value of <.05 was considered significant.

Crude estimates of age-specific incidence rates were calculated for each 5-year age groups (0–4 years, 5–9 years, 10–14 years, and 15–19 years) as the quotient of the number of cases and the total number of persons in each sex and geographical area based on recent population projections by the year 2022 (**Supplementary Table 1**) (2, 11). The overall crude incidence rates (CIR) were calculated as the weighted averages of the respective age-specific incidence rates in each age range for 0-14 years and 0-19 years respectively. All the crude rates were adjusted to the world standard population in order to derive the overall age-standardized rates (ASR) for the population (13).

### Ethical considerations

Ethical approval for the study was obtained from the Health Research and Ethics committee of Federal Medical Centre, Azare, Bauchi State.

## Results

### Demographic characteristics of children and adolescents in study sites

Children and adolescents aged 0-19 years in the four participating states of the northeast geopolitical zone had a combined population of 17,178,825 (**Supplementary Table 1**). 50.6% of these are males while 49.4% are females.

### Childhood cancer demographics

A total of 346 cancer cases in children and adolescents were included in the analysis after exclusion of cases with incomplete demographic data, no specification of cancer location or cases that were inconsistent with the diagnosis of cancer. Over the same study period, a total of 4681 confirmed cancers were diagnosed in the region resulting in a proportion of pediatric cancers of 7.3%. There were 206 males and 140 females with an incidence sex ratio (male to female) of 1.47:1 that was statistically significant (p=0.001; χ^2^ = 114).

The overall crude incidence rates (CIR) for childhood cancers were 20.9 per million (for 0-19 years) and 18.8 per million (for 0-14 years) respectively. The overall age-standardized incidence rates (ASR) for childhood cancers in this study was 7.21 per million (0-19 years) and 4.90 per million population (0-14 years) respectively. The respective ASR for males and females aged 0-19 years were 8.43 and 5.97 per million population, respectively.

**Figure 1:**
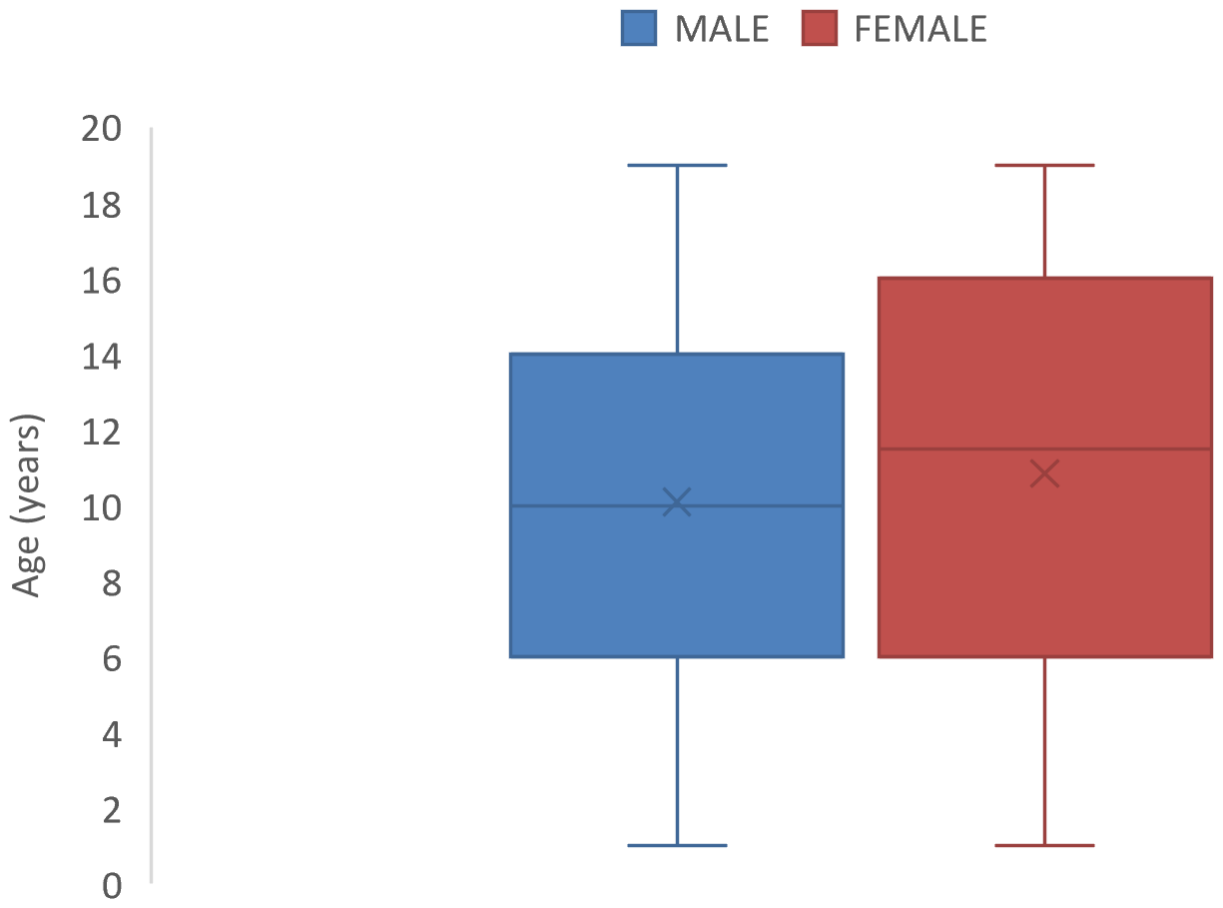
Box and Whisker plots showing median ages (and interquartile ranges) of male and female children with Childhood cancer in North-East Nigeria, 2019-2022.

The median age at diagnosis was 10.5 years {inter-quartile range (IQR)= 6-16years} with a slightly earlier median age of diagnosis for males (**Figure 1**).

The crude and age-standardized incidence rates (ASR) for cancers in each 5-year age group is shown in **Table 1**. The highest absolute proportion of cases were found in the 10-14 years age group; however, the age group 15-19 years had the highest ASR of 2.31 per million population.

**Table 1:** Incidence rates of childhood cancers in North East Nigeria (2019-2022).

Age group (years)	No. of cancer cases (%)	Crude age-specific incidence rates (per million person-years)	Age-standardized rates (per million population)
0-4	63 (18.2)	12.3	1.09
5-9	88 (25.4)	19.6	1.70
10-14	102 (29.5)	24.5	2.11
15-19	93 (26.9)	27.3	2.31
Total	346 (100.0)		7.21

**Figure 2:**
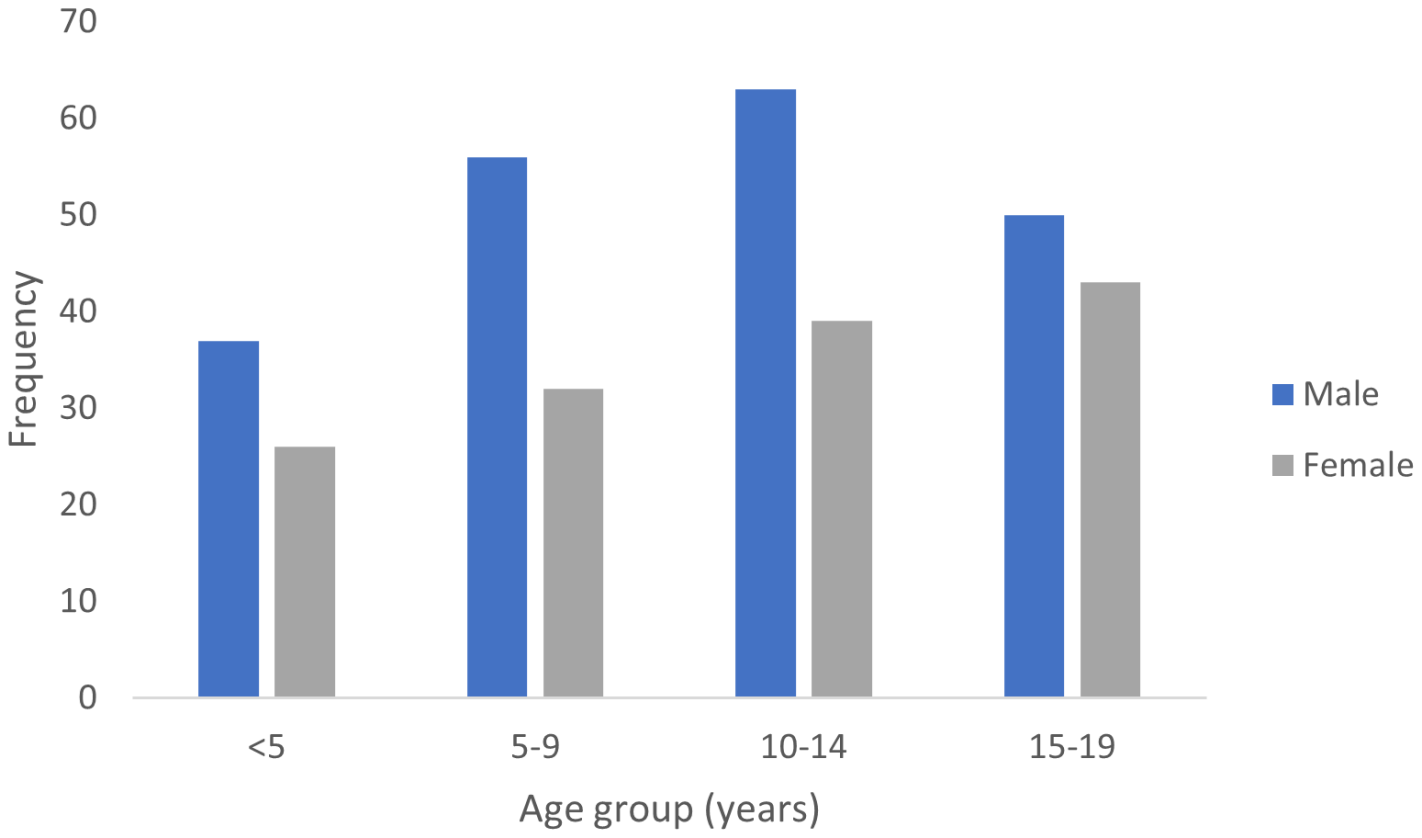
Age group distribution of childhood cancer cases in North-East Nigeria, 2019-2022.

Cumulatively, children aged 0-14 years constituted 73.1% (253 cases) while adolescents aged 15-19 years constitute 26.9% of cases. There was a significant variation in cancer incidence across age groups (p < 0.0001, Cramér’s V = 0.45). The peak age range at diagnosis of pediatric cancers within the period under review was 10-14 years (**Figure 2** **).** However, the 15-19 years age group had the highest ASR as shown in **Table 1**.

### Diagnostic grouping of childhood cancer cases

The distribution of the cancers by ICCC-3 diagnostic groups is presented in **Table 2**. Lymphomas (23.4%) constitute the most commonly diagnosed childhood cancers while CNS neoplasms (0.6%) were the least frequently diagnosed cancers.

**Table 2:** The Distribution of childhood cancers in North East Nigeria (2019-2022).

Diagnostic Group	Frequency (%)	Median Age (minimum-maximum) in years	Male: Female ratio
I. Leukemia, MPD & MDS *ALL* *AML* *Chronic MPDs* *Unspecified leukemia* Subtotal	20 (5.8) * 9 (45.0)* * 7 (35.0)* * 2 (10.0)* * 2 (10.0)* 20 (100.0)	8.5 (3-18) 8 (4-15) 13 (7-17) - -	5.6:1 - - - -
II. Lymphomas and RE neoplasms *Hodgkin* *Non-Hodgkin, non-Burkitt* ***Burkitt*** * Unspecified* Subtotal	81 (23.4) *30 (37.0)* *26 (32.2)* *24 (29.6)* *1(1.2)* * 81(100.0)*	11 (4-19) 12.5 (6-19) 11 (4-18) 8 (4-14) -	2.7:1 5:1 3.3:1 1.2:1 -
III. CNS neoplasms	2 (0.6)	5.5 (3-8)	–
IV. Neuroblastoma	22 (6.3)	3 (1-17)	1:1
V. Retinoblastoma	24 (6.9)	5.5 (1-14)	1.4:1
VI. Renal tumors *Nephroblastoma* *Renal carcinomas* *Unspecified* Subtotal	41 (11.8) *39 (95.2)* *1 (2.4)* * 1 (2.4)* 41 (100.0)	5 (1-17) 5 (1-13) - -	1.4:1 1.4:1 - -
VII. Hepatic tumors *Hepatoblastoma* *Undifferentiated embryonal sarcoma* Subtotal	3 (0.9) *1 (33.3)* *2 (66.7)* 3(100.0)	11 (2-24) - -	- - -
VIII. Malignant bone tumors *Osteosarcoma* *Chondrosarcoma* *Round cell sarcoma, NOS* Subtotal	28 (8.1) * 26 (92.8)* *1 (3.6)* *1 (3.6)* 28 (100.0)	17 (3-18) 17 (10-19) - -	2:1 2.1:1 - -
IX. Soft Tissues and other extra-osseous neoplasms *Rhabdomyosarcoma* *MPNST* *Synovial sarcoma* *Kaposi sarcoma* *Alveolar soft part sarcoma* *Liposarcoma* *Unspecified round cell & spindle cell sarcomas* Subtotal	50 (14.5) * 36 (72.0)* * 2(4.0)* * 3 (6.0)* *2 (4.0)* * 1 (2.0)* *1 (2.0)* *5 (10.0)* 50 (100.0)	12 (1-19) 10 (1-19) - - - - - -	1.2:1 2:1 - - - - - -
X. Germ cell, trophoblastic & gonadal neoplasms *Extragonadal germ cell tumors* *Gonadal germ cell tumors* *Gestational choriocarcinoma* Subtotal	8 (2.3) *2 (25.0)* *5 (62.5)* *1 (12.5)* 8 (100.0)	15.5 (5-19) - - -	1:7 - - -
XI. Other Malignant epithelial neoplasms and melanomas *Thyroid carcinoma* *Nasopharyngeal carcinoma* *Cutaneous carcinoma* *Salivary gland carcinoma* *Colorectal carcinoma* *Ocular carcinoma* *Breast carcinoma* *Esophagus* *Urinary bladder* *Cervix* Subtotal	50 (14.5) *1 (2.0)* *10 (20.0)* *17 (34.0)* *4 (8.0)* *6 (12.0)* * 3 (6.0)* *6 (12.0)* *1 (2.0)* *1 (2.0)* *1 (2.0)* 50 (100.0)	15 (3-19) - 16.5 (4-19) 14 (3-19) - 17 (14-18) - 16.5 (7-18) - - -	1:1.2 - 1:1 - - 1:5 - - - - -
XII. Other and Unspecified Malignant Neoplasms	17 (4.9)	16 (4-18)	0.89:1
Total	346		

ALL, Acute lymphoblastic leukemia; AML, Acute myeloblastic leukemia; CNS, Central nervous system; MPD & MDS, Myeloproliferative disorders and Myelodysplastic syndrome; MPNST, Malignant peripheral nerve sheath tumor; NOS, Not otherwise specified; RE, Reticuloendothelial.

Overall, the top 5 commonly diagnosed cancers in the age 0-19 years in descending order of frequency includes Lymphomas (23.4%); Soft tissue sarcomas (14.5%); Malignant epithelial neoplasms (14.5%); Malignant renal tumors (11.8%) and Malignant bone tumors (8.1%). However, in children aged 0-14 years, the top 5 commonest cancers were lymphoma (26.9%); renal cancer (15.8%), soft tissue sarcoma (15.4%), retinoblastoma (9.5%) and epithelial cancers (8.7%). The corresponding top 5 commonest cancers in adolescents aged 15-19 years were epithelial cancers (30.1%), Bone cancers (21.5%), lymphoma (14.0%), soft tissue sarcoma (11.8%) and other unspecified malignant neoplasms (10.8%).

### Age group characteristics

**Figure 3:**
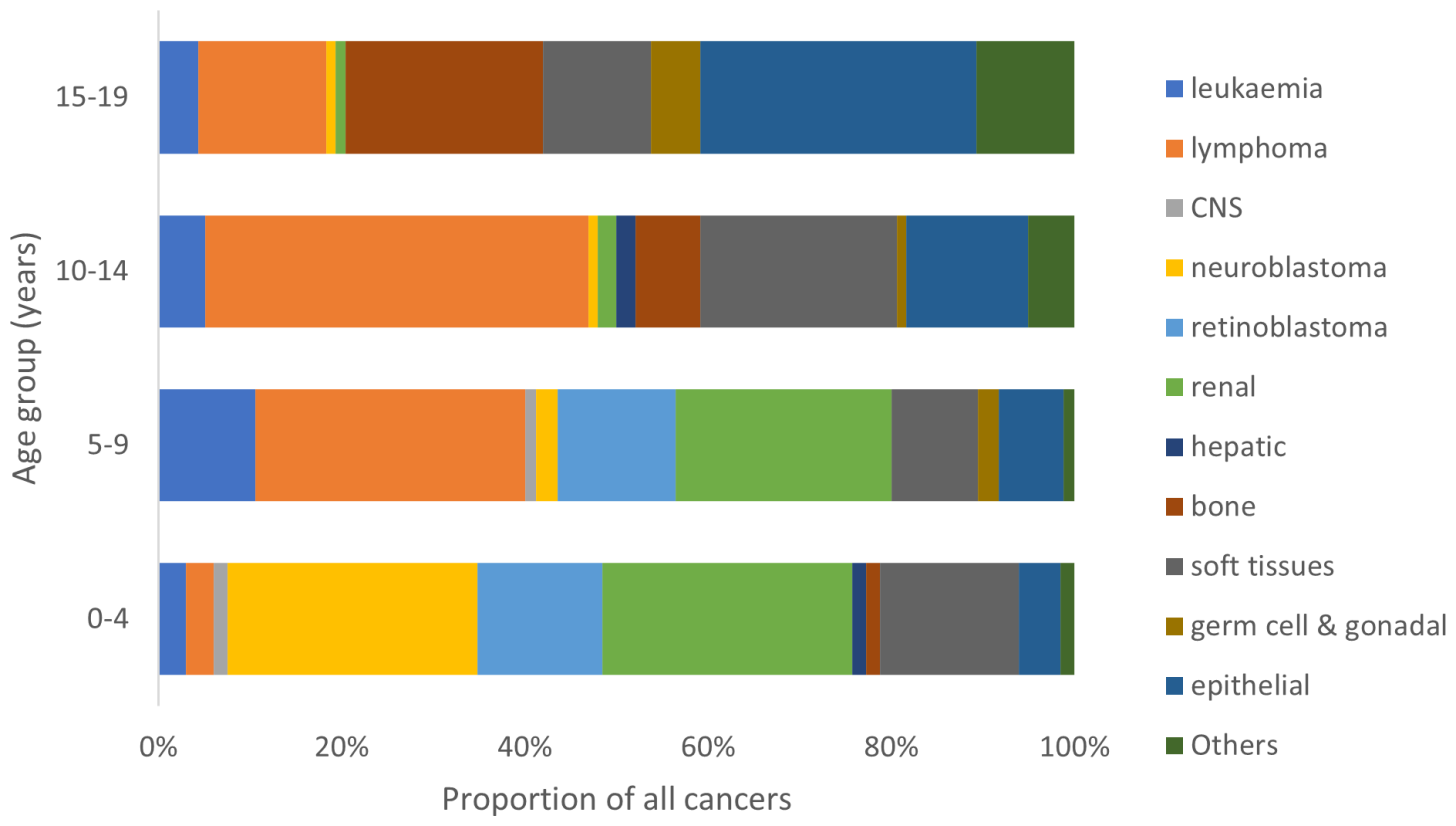
Proportional distribution of cancer types by age group of children in North-East Nigeria, 2019-2022.

There was a remarkable variation in the range of cancer types across different age groups (**Figure 3**). In the age group from birth to 4 years, neuroblastoma and renal tumors (mainly nephroblastoma) were the predominant cancers. Lymphoma, renal tumors and retinoblastoma predominated in the 5-9 years age group while lymphoma and soft tissue sarcomas constituted the most common cancers in the 10-14 years age group. Carcinomas, bone cancers and lymphoma were the most common tumors diagnosed in the 15-19 years age group.

#### Lymphomas

**Figure 4:**
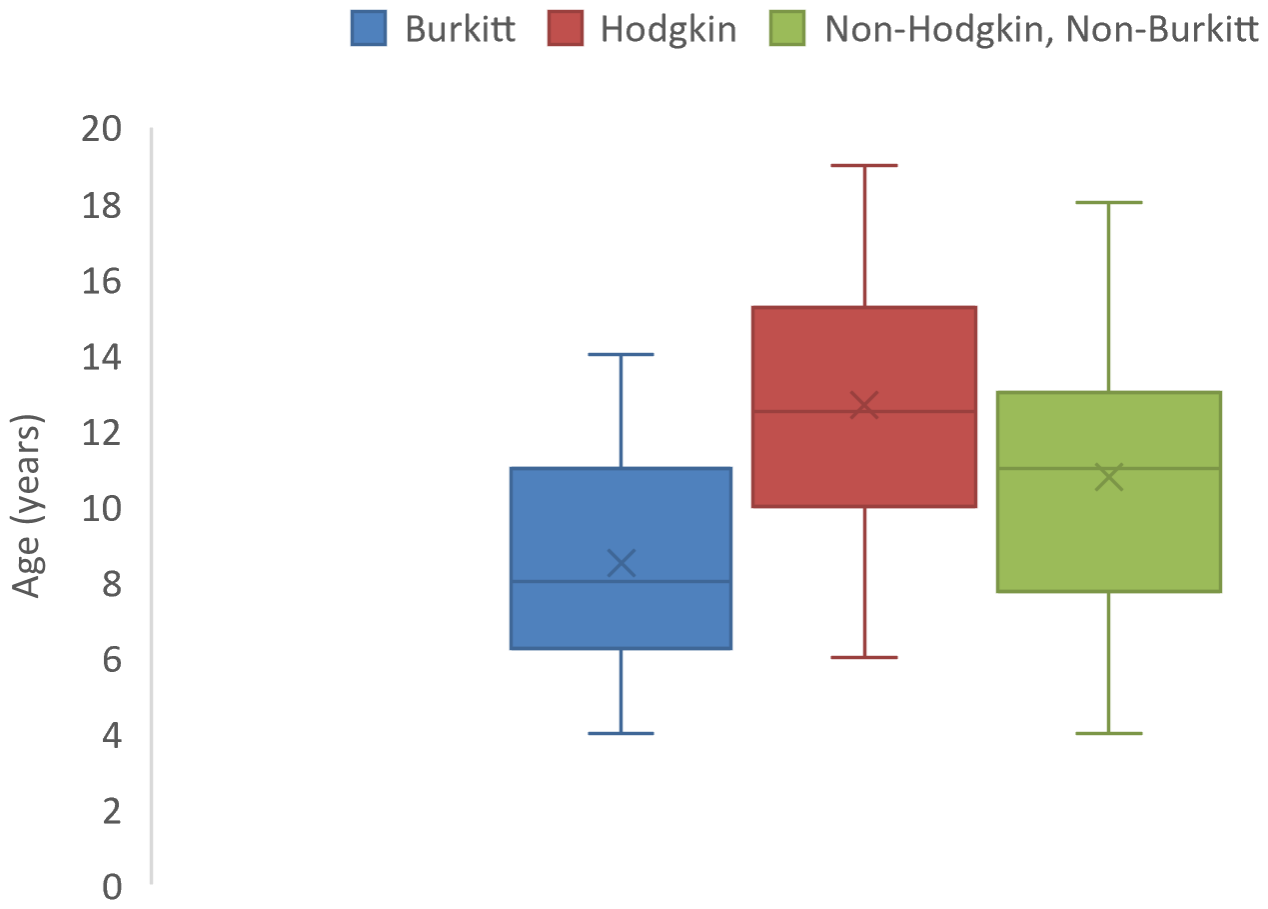
Box and whisker plot showing median ages (and interquartile ranges) of lymphoma cases of children in North-East Nigeria, 2019-2022.

Lymphomas constituted the most frequently diagnosed childhood cancers with a median age at diagnosis of 11 years (IQR: 8-13 years) and a male: female ratio of 2.7:1 (**Table 1**). Hodgkin lymphoma was the most commonly diagnosed lymphoma constituting 37.0% of all lymphomas. They occurred at a median age of 12.5 (IQR: 10-15.3) years (**Figure 4**). All the cases in this study occurred in the cervical lymph nodes. Non-Hodgkin lymphomas (excluding Burkitt) constituted 32.1% of all lymphomas with median age at diagnosis of 11 (IQR: 7.8-13) years (**Table 1**). Burkitt lymphoma constituted 29.6% of lymphomas diagnosed in this study and occurred at a median age of 8 years (IQR: 6.3-11 years) as shown in **Figure 4**. Majority (87.5%) of the Burkitt lymphomas occurred in extra-nodal locations with the jaw (58.3%) being the most common site of occurrence, followed by intra-abdominal locations (20.8%).

#### Malignant soft tissue tumors

Soft tissue sarcomas were the 2^nd^ most commonly diagnosed malignancies of childhood and adolescence constituting 14.5% of all cancers in these age groups with a median age at diagnosis of 12 (IQR: 5-14) years (**Table 2**). An overwhelming majority of the tumors were rhabdomyosarcomas (72.0%).

#### Malignant epithelial neoplasms

Malignant epithelial neoplasms were the joint 2^nd^ commonest group of neoplasms in this study accounting for 14.5% of all cancers. The median age at diagnosis was 15 (IQR: 12.8-17) years (**Table 2**) and cutaneous carcinomas were the most commonly diagnosed cancers in this diagnostic category.

#### Malignant renal tumors

Renal malignancies constituted the 4^th^ most common group of cancers in children with a median age at diagnosis of 5 (IQR: 3-8) years (**Table 2**). Majority of these malignancies were nephroblastoma with only occasional cases of renal carcinomas (**Table 1** **).**

#### Malignant bone tumors

Bone cancers constituted the 5^th^ most commonly diagnosed malignancy accounting for 8.1% of all malignancies in childhood and adolescence in this study. The median age at diagnosis was 17 (13-18) years (**Table 2**), and roughly 93% of these cancers were osteosarcoma.

## Discussion

With the aid of available data from the contributing tertiary health institutions in North-East Nigeria, we report the estimated incidence rates of cancer in children aged 0-19 years between 2019-2022: 20.9 per million children (0-19 years) and 18.8 per million children (0-14) years respectively. The respective ASR were 7.21 and 4.90 per million. These values are far lower than overall ASR estimates for sub-Saharan Africa (50-150 per million) reported by Steliarova-Foucher et al. in the IICC3 (2). This obvious discrepancy is not altogether surprising considering that the IICC3 report is based on data from high quality population-based cancer registries, which was generally lacking in our study. Because most of our data were from institution-based departmental registers, there is likely to be an under-estimation of cancer cases with less likelihood of data quality assurance. In addition, only microscopically confirmed cancer cases were included in this study. Previous population-based registry reports in Africa have revealed varying ASR for childhood cancers across the continent from as low as 26.8 per million children in The Eastern cape of the Republic of South Africa (2003-2012), to as high as 308.2 per million children in Blantyre, Malawi (2003-2010) (14). An ASR of 80.6 per million was also previously reported from the Ibadan cancer registry, Nigeria (2003-2012). The strong possibility of under-estimation of childhood cancer cases in this study underscores the importance of establishing and strengthening population-based cancer registries in the North East and other parts of Nigeria.

The proportion of childhood malignancies in this study in relation to the total number of malignancies over the study period (7.3%) falls within the range of 1.4-10% reported by most studies within the sub-Saharan African sub region (5, 15–17). It is noteworthy that most of these studies were on cancers occurring within the age ranges of 0-14 years in contrast to our study in which the age range is extended to 19 years. In this study, if only the age group 0-14 years is considered, then the proportion of childhood cancers drops down to about 5.4% of all cancers over the study period.

This study revealed a median age at diagnosis of 10.5 years. One institution-based study in Nigeria reported a median age of 4.75 years at diagnosis (18). However, the study collected cancer data from children less than 15 years. In other parts of sub-Saharan Africa, a study based on two (2) population-based cancer registries in Zimbabwe and Uganda that collected cancer data for people aged 0-19 years, reported an overall median age of occurrence of 9 years (19). Another study at a Ugandan Cancer Institute reported a median age at diagnosis to be 7 years among people aged 0-19 years (20).

The slightly higher incidence of the cancers in boys (sex ratio 1.47:1) is in keeping with most other national and continental studies of pediatric cancer as well as global estimates (2, 7, 15, 18). The reasons why cancers are commoner in boys than girls remain elusive. Boys and girls share similar genetic and environmental risks for childhood cancers and are therefore expected to have similar cancer incidence. However, some authors have observed in very early studies that male children are more likely to be sent to receive specialist medical care in countries with low gross domestic product (GDP), due to prevailing cultural practices that favors the male child (21, 22). There is currently no evidence to suggest that our finding reflects gender discrimination in health-seeking behavior. However, this possibility may need to be examined in more detail in other studies.

Lymphomas were the most frequently diagnosed childhood malignancies with a median age at diagnosis of 11 years. This is in keeping with previous observations in Nigeria and other parts of Africa (13–16, 20). However, in contrast to most previous studies where Burkitt lymphoma constituted the predominant lymphoma subtype, Hodgkin lymphoma and non-Hodgkin, non-Burkitt lymphoma both outnumbered Burkitt lymphoma in our series. This may be due to the extension of the study participants to 19 years in this study whereas other studies covered only the 0-14 years age range and the fact that endemic Burkitt lymphoma rarely occurs beyond the age of 17 years. Despite the wider age range, the median ages of occurrence of all the 3 lymphoma subtypes were <15 years (Hodgkin, 12.5 years; non-Hodgkin non-Burkitt, 11 years; and Burkitt, 8 years). It would appear that there is a changing pattern in the occurrence of various lymphoma subtypes, however more large-scale studies on the subject are required before such conclusions can be drawn.

Leukemia is considered to be the most common cancer of children worldwide (2). However, our study, just like most other African studies did not reveal a preponderance of leukaemia (15, 16, 20). A notable exception is a 4-year Ghanaian series in which leukemias were the 2^nd^ most commonly diagnosed cancer in childhood (23). In this study, it is the 6^th^ and 7^th^ most common cancer in the age ranges 0-14 years and 15-19 years respectively and did not feature as a top 5 cancer in any age group except for the age 5-9 years where it ranked 4^th^. Underdiagnosis/misdiagnosis of leukemia due to inadequate diagnostic and treatment facilities, is often touted as the likely reason for the low incidence of leukemia in African populations (24, 25). It is however worthy of note that a low leukemia incidence has also been observed among African American children of the USA in comparison to other ethnic groups (2). While the possibility of certain genetic alleles conferred by the common African ancestry may be a factor in this observation, it may also have resulted from reduced access to health care as a result of lower socioeconomic status of this population group (26, 27).

Soft tissue sarcomas (mainly rhabdomyosarcomas) were a significant contributor to childhood cancer burden in this study and was the 2^nd^ only to lymphoma as the most common cancer in people aged 0-19 years. This finding is in agreement with data from the cancer registry of Ibadan, Nigeria (2003-2012) where soft tissue sarcoma (predominantly rhabdomyosarcoma) was the 2^nd^ commonest cancer after lymphoma (14). The data from other parts of Africa show varied results that range from being the 3^rd^ commonest childhood cancer in Ethiopia and Kenya to not being among the top 5 paediatric cancers in most other countries (14, 17). It is noteworthy that national cancer registry data from Zambia and Zimbabwe show that Kaposi sarcoma is the most commonly diagnosed soft tissue sarcoma rather than rhabdomyosarcoma observed from other African countries (17). This may reflect the effect of HIV prevalence and morbidity in these locations.

As expected, epithelial cancers (carcinomas) were the most commonly diagnosed cancers (30.1%) in the 15-19 years age group, but it was also a relatively significant cancer type in the 10-14 years age group (12.7%) where it was the 3^rd^ most commonly diagnosed group of cancers. An overwhelming majority of these carcinomas were cutaneous squamous cell carcinoma, many of which were related to chronic scars and discharging sinuses. In the much younger age groups, carcinoma often occurred in exceptional circumstances such as the occurrence of rare cutaneous and conjunctival carcinomas in association with xeroderma pigmentosum (28, 29). In general, it appears that the incidence of carcinomas in childhood increases with the age of the child.

A striking observation in this study is the paucity of cases of CNS neoplasms, which is one of the major neoplasms occurring in children in high-income countries (2). As has been previously observed, the lower incident cases of CNS neoplasms in children of low and middle income countries (LMICs) likely reflect the paucity of paediatric neurosurgical and neuro-imaging facilities and personnel (2, 30).

The IICC3 category XII titled ‘Other and Unspecified malignant neoplasms’ constitute about 4.9% of all cancers in this study. A significant proportion of the cancers in this group were “malignant small round blue cell tumors” and metastatic carcinomas of undetermined origin. This implies that at least some of the cancers in this category reflects limitations in specialized and ancillary diagnostic facilities required for specific characterization of these malignancies.

### Limitations

Notable limitations were inherent in this study, mostly affecting data accrual and quality. As a hospital cancer registry-derived data, the cases presented here reflect the available skill capacity and infrastructure available in the region, and not necessarily a true reflection of the disease burden in the index population. Likewise, systematic follow up of the patients was not documented, hence information on therapy, survival and mortality is also lacking in the study. Additionally, not all the states in the zone had their data included in this analysis, hence, caution is required in extending our findings to the entire northeast region. Future studies should therefore incorporate clinically diagnosed cancers, cover the entire geographical area and search for survival data that could elucidate on what therapy options are more likely to be effective in the population, while suggesting preventive strategies.

## Conclusion

This study provides useful insights into the pattern of occurrence of childhood cancers in this region and can serve as a baseline for more robust studies in the country. There is an urgent need to upscale existing infrastructure, as well as establish and/or strengthen population-based cancer registries in the region and country at large so as to ensure the availability of high-quality data that will be invaluable to childhood cancer control efforts. There is also a need to improve capacity-building in the diagnosis and management of childhood cancers in our environment to ensure that the number of ambiguous or non-specific diagnoses are markedly reduced.

## Data Availability

The raw data supporting the conclusions of this article will be made available by the authors, without undue reservation.
