# Supplementary material for: Incidence of childhood cancers in the North East geopolitical zone of Nigeria
Source: Front Oncol. 2024 Aug 30;14:1379968. doi: 10.3389/fonc.2024.1379968 (PMC11392771; doi:10.3389/fonc.2024.1379968)
Supplement: Supplementary file 1 [file Table1.docx]

Supplementary Table 1: Age group by gender among the population of Children and Adolescents in 4 states of NE Nigeria

| **Age group** | **Male** | **Female** | **Total** |
| --- | --- | --- | --- |
| **0-4** | 2,598,386 | 2,524,709 | 5,123,095 |
| **5-9** | 2,263,043 | 2,224,031 | 4,487,074 |
| **10-14** | 2,095,811 | 2,068,171 | 4,163,982 |
| **15-19** | 1,738,137 | 1,666,537 | 3,404,674 |
| **Total** | 8,695,377 | 8,483,448 | 17,178,825 |
